# Meat Analog Products: Current Worldwide Scenario and Future Perspectives in Consumption and Regulation

**DOI:** 10.3390/foods15020376

**Published:** 2026-01-20

**Authors:** Tatiana Barbieri Cochlar, Ziane da Conceição das Mercês, Natalia Maldaner Salvadori, Sabrina Melo Evangelista, Virgílio José Strasburg, Viviani Ruffo de Oliveira

**Affiliations:** 1 Postgraduate Program in Food, Nutrition, and Health, Faculty of Medicine, Federal University of Rio Grande do Sul (UFRGS), 2400 Ramiro Barcelos St., Porto Alegre 90035-003, Brazil; tatianabarbieri2010@hotmail.com (T.B.C.); zianemerces@gmail.com (Z.d.C.d.M.); natisalvadori18@gmail.com (N.M.S.); sabrina.evangelista@ufrgs.br (S.M.E.); virgilio_nut@ufrgs.br (V.J.S.); 2 Department of Nutrition, Federal University of Rio Grande do Sul (UFRGS), Porto Alegre 90035-003, Brazil

**Keywords:** meat substitutes, food consumption, food regulations, food labeling, sustainability

## Abstract

Interest in plant-based diets has grown expressively in different regions of the world. However, the missing regulation for meat analogs may mislead consumers by suggesting that these products are the same as the meat they are replacing. Therefore, this study aims to analyze the current global scenario of meat analogs, discuss consumption changes and their regulation, as well as pointing out future perspectives for the sector. A narrative literature review was performed using scientific papers from the Virtual Health Library (BVS), LILACS, PubMed (NIH), Embase, Web of Science, Scopus, and official documents. Included studies were aligned with the research theme, concentrating on countries with regulations for plant-based analog products and those lacking or pursuing such regulations. Additionally, studies were selected based on the following criteria: original or review studies from different countries, papers discussing meat analogs in terms of consumption, sensory attributes, market dynamics, sustainability, regulation, food safety; availability of full text; and publication dates ranging from 2015 to 2025. The data reveals that most of the assessed nations still lack specific regulations for meat analog products, adopting general labeling and naming standards that range from flexible approaches to strict restrictions. To conclude, the article highlights that meat substitutes are emerging as promising and sustainable options; however, their true consolidation is conditioned on the existence of more defined regulatory frameworks, increased consumer confidence, and market conditions that favor their large-scale adoption.

## 1. Introduction

Meat and its by-products play a central role in global food culture, with consumption patterns influenced by cultural, religious, economic, and sustainable factors [1,2], as well as valued sensory attributes, convenience in preparation, contribution to conservation strategies, and waste reduction [2,3,4]. According to the Planetary Diet, the recommended intake of animal meat is 14 g/day for beef, lamb and pork; 29 g/day for chicken and other poultry; and 28 g/day for fish. However, most countries exceed this guideline by more than five times [5]. Projections indicate a further increase in meat production driven by economic growth, urbanization, and rising incomes in developed countries [6].

On the other hand, there is also a growing interest in plant-based diets in different regions of the world. In Italy, a study showed an increase in the intention to reduce meat consumption [7]. In the United States for example, about two-thirds of consumers reported reducing their meat consumption in recent years as well [5,8,9]. Alcorta et al. [8], using statistical data from Lantern—The Green Revolution [10], stated that in the United Kingdom, 21% of the population identify themselves as flexitarians, that is, those who follow a vegetarian diet, but occasionally consume animal products. Moreover, one out of eight individuals claimed to be vegetarian or vegan. In Germany, the proportion of vegetarians rose from 1% in 2005 to 7% in 2018, while in Spain, flexitarians increased by 25% over two years [10]. In addition, there are flexitarians who sympathize with the cause, and there are also those who simply decide to increase their consumption of plant-based foods in their routine because they want to eat more of these food groups.

In line with the reduction of meat consumption for health reasons, there is a global movement to make the food system more sustainable by reducing the use of products with high greenhouse gas (GHG) emissions [11]. The environmental and health impact of consuming animal-based foods has encouraged the search for plant-based alternatives [12,13]. And this change in consumption has proven to be significant, since the production of animal-based foods generates about one-third of GHG emissions, uses 40% of the land, and consumes more than two-thirds of fresh water [14], in addition to involving ethical and health aspects [15].

Meat alternatives encompass various products, including meat analogs [16,17], lab-grown meats [18], and insect-based foods [19], all designed to facilitate the shift towards a more sustainable food future [11]. Their purpose is to replicate the visual effect, culinary expectations, and all the sensory attributes of the authentic meat products, such as hamburgers [20,21,22], ground meat [23], meatballs [24], and sausages [25].

Although meat analogs have a history of consumption in various markets, the lack of standardization in production processes and nomenclature continues to be an obstacle to product comparability and the transparency of consumer information. These factors can impact the progress of innovation and global competitiveness [26]. The absence of specific regulations can compromise food and legal assurance, as well as lead to variations in the nutritional composition of these foods [27].

In this context, regulatory policies should seek to achieve a balance, so that standards are harmonized and consistent across countries, without limiting technological development in the sector [28,29]. Therefore, this study aims to analyze the current worldwide scenario of meat analog products, discuss consumption changes, and regulatory aspects, as well as pointing out future perspectives for the sector.

## 2. Methodology

This integrative literature review was conducted between February of 2024 and November of 2025, through the analysis of official documents and scientific articles available in the following databases: Virtual Health Library (BVS), Latin American and Caribbean Health Sciences Literature (LILACS), National Library of Medicine (NIH) PUBMED, Embase, Web of Science, and Scopus, as well as official documents.

As an inclusion criteria, all the documents must be related to the central theme of the research and include the G20 countries, besides had to meet the following requirements: (1) original articles—quantitative or qualitative, systematic reviews, meta-analyses, and relevant narrative reviews; (2) studies addressing meat analog products in terms of consumption, sensory acceptance, market, sustainability, and regulation; (3) accessibility—articles available in full for reading; (4) documents published from 2015 to 2025.

The selection of the G20 countries was made for convenience, justified by the ABC curve method, which classifies information by distinguishing items of greater importance or impact from those typically in smaller numbers or less representative [30]. In this investigation, the G20 countries represent about 85% of the Gross Domestic Product (GDP), more than 75% of global trade, and around two-thirds of the world’s population. Other studies that evaluate the use of raw materials and environmental impacts have already used the representativeness of the method [31,32,33].

Exclusion criteria were: (1) duplicate articles or secondary versions (e.g., preprints that have already been published); (2) papers that do not present complete data or are not available in full; (3) studies that only address conventional meats (animal-based) without focusing on meat analogs; (4) publications such as conference abstracts, editorials, letters to the editor, and opinions without scientific foundation; and (5) articles outside the thematic scope (e.g., those that deal with vegetarianism without relation to meat analog products).

The keywords used for this review article were based on Medical Subject Headings (MeSH), EMTREE, and Health Sciences Descriptors (DeCS), combined with the Boolean operators “OR” and “AND” as shown in Table 1.

**Figure 1:**
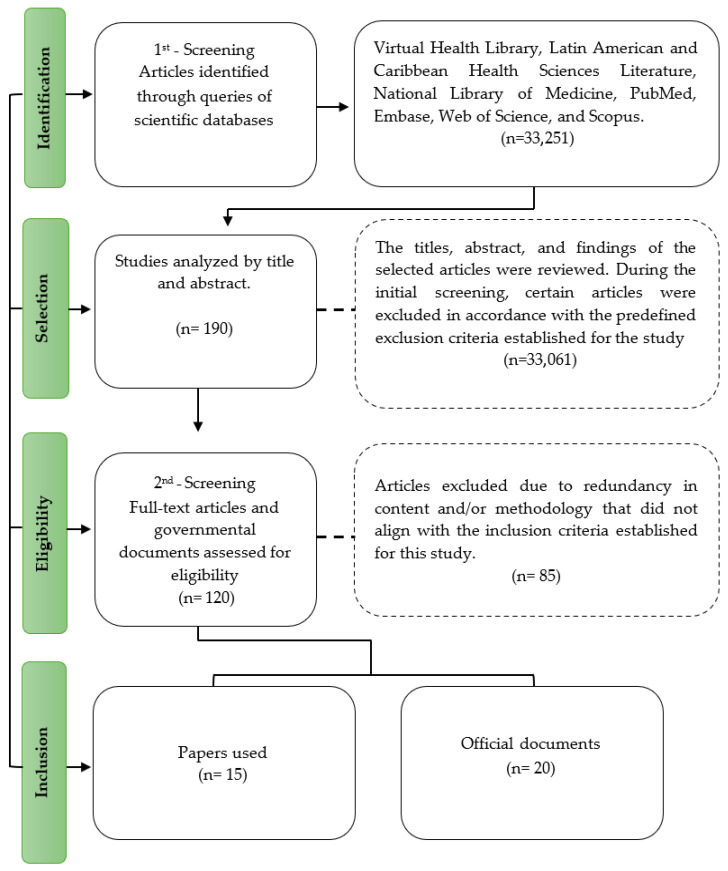
Flowchart of the selection and inclusion of papers and official documents analyzed. Source: Study data.

The articles were selected by first reading the titles and their abstracts, as well as official documents from websites related to the subject, to verify their relevance to the study. Subsequently, the documents that met the inclusion criteria were read in entirety (Figure 1).

After the selection process, which included reading titles and abstracts followed by evaluation of the full text, several scientific articles [n = 15] met the eligibility criteria and were included in this narrative review. In addition, official regulatory documents [n = 20] from international organizations were selected to support the analysis of regulatory frameworks, labeling standards, and food safety aspects related to meat-analog products.

## 3. Changes in Adherence to Plant-Based Diets

Changes in consumption patterns related to decreased meat consumption and increased adoption of plant-based diets are evidenced in studies with diverse populations and cultures, as presented in Table 2. The findings indicate a continuous growth in adherence to plant-based dietary patterns, motivated by the pursuit of improved nutritional quality of diets, driven by the greater availability of alternative products to meat in the market and changes in consumer perceptions of these products.

In the United States, a longitudinal study showed a consistent increase in the adoption of plant-based dietary patterns in recent decades, evidencing that this increase in consumption proportionally influences the decrease in the intake of animal proteins in the diet [34].

In Europe, it can be seen that in the German population, the pleasure associated with the consumption of meat products represents a limiting factor for this reduction [35]. On the other hand, research carried out in Italy [36] and Poland [37] indicated that the intention to reduce meat consumption is closely related to the previous frequency of plant-based food intake, environmental identity and negative feelings towards meat.

In addition, public policies, marketing strategies, and labeling are relevant variables in the acceptance and selection of plant-based alternatives. Observational studies and population surveys in the European context have shown that a significant proportion of consumers express an intention or effective practice of reducing meat consumption, mainly motivated by health-related issues [36,37].

## 4. Regulation for Meat Analogs

The regulation of meat analogs represents one of the main challenges in the consolidation of this food sector on a global scale. Despite presenting similarities in terms of appearance, functionality, and sensory characteristics with conventional meat products, these foods must also ensure an equivalent nutritional composition, as their labeling often directly mentions animal-derived products [40].

It is well known that labels influence perception and categorization processes [41]. In the food sector, traditional regulation focuses on consumer protection [42], but studies have emphasized the need for regulatory frameworks to enable the innovation process and avoid the loss of benefits and potential that food innovations should offer [41,42,43]. According to Demartini et al. [41], labels induce consumers to make inferences about properties or attributes that, although not directly observable, should be possessed by a target stimulus.

Thus, the actions of political and regulatory agents are vital for preserving legitimacy and equity in the process of transforming food systems. As emphasized by Lähtteenmäki-Uutela et al. [42], it is important that all stakeholders in the system, including producers, traders, and consumers, participate in the transition to ensure the implementation of consistent and fair food policies.

In the global context, differences are observed in the regulatory standards concerning meat analogs. Among the G20 countries, as well as in the African Union (AU) and the European Union (EU).

**Figure 2:**
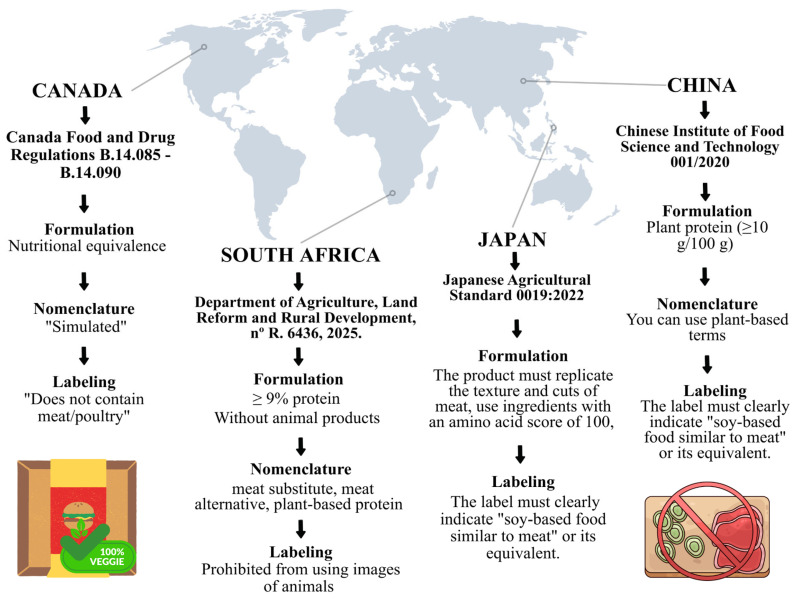
Guidelines regarding the formulation, nomenclature, and labeling of meat analog in regulated nations (Canada, China, Japan, and South Africa). Source: Prepared by the author (2026).

It is noted that only a few nations (n = 4), South Africa, Canada, China, and Japan, have specific regulations directed at these products, as evidenced in Figure 2. Although only these countries have specific legislation already published for their products, it is worth noting that the subject is under careful consideration in many countries, encouraging new research and official documents for upcoming publications. This would lead to the standardization of meat analog products, ensuring consumer confidence and encouraging the transition to a sustainable diet, as well as regulation of products, which is also extremely important for the meat industry and producers. This finding also demonstrates the diversity of food policies around the world and highlights the urgency of regulatory harmonization among major economies and regions.

It is evident that countries like China and Japan allow the use of “plant-based” designations if they are accompanied by technical requirements of sensory or nutritional similarity, reinforcing the link between product authenticity and informational clarity. According to Zhang et al. [26], despite the safe use of meat analogs, discrepancies regarding nomenclature and product standards still limit innovation and global trade, requiring clear regulations on naming and technical criteria.

In addition to the previous recommendations on the labeling of meat analogs, Visagie [44] suggests that, to reduce the risk of consumers being misled, the information “plant-based” and “does not contain meat” should be presented clearly and prominently on the front of the packaging, ensuring transparency about the composition of these products.

Although a minority of countries have specific legislation for meat analogs, the Codex Alimentarius (CXS 174-1989, Rev. 2022) [45] establishes the “General Standard for Plant Protein Products”, guiding the safe and appropriate use of these products, ensuring nutritional quality (of at least 40% protein) and proper labeling. This regulation allows for the recommendation of partial or total replacement of animal proteins, provided they maintain nutritional equivalence in terms of the quantity and quality of proteins, vitamins, and minerals [38,45].

In addition, the International Organization for Standardization (ISO) has issued standard ISO 23662:2021, [46] which delineates definitions and technical criteria for foods and food ingredients deemed suitable for vegetarians or vegans, as well as for labeling and claims. This standard stipulates explicit criteria for labeling and nutritional assertions, forbidding the inclusion of any animal-derived food or ingredient, encompassing additives, technological auxiliaries, flavors, and enzymes. ISO 23662:2021, however, does not establish formulation standards for products or nutritional sufficiency concerning food substitutions.

Therefore, these international standards are often mentioned as starting points for international regulatory harmonization, although they do not yet establish specific nutritional criteria for products intended to replace foods of animal origin [27]. It can be seen that the legislation found provides guidelines on nutritional adequacy requirements and recommends the exclusion of animal-based foods from their formulations, in accordance with the regulations mentioned.

Most of the countries analyzed still show flaws in their specific regulations regarding meat analog products, limiting themselves to broad and poorly detailed legislation for this emerging category in the food sector (Table 3). This regulatory gap highlights the speed of technological innovation, in contrast to the slowness of normative processes, which continue to be linked to traditional models of animal protein production [43].

The comparative analysis of the legislation reveals significant heterogeneity in the approaches, especially regarding the nomenclature, labeling, and formulation of these products. In nations such as France and Italy, a restrictive approach is noted, with an explicit ban on the use of terminology traditionally associated with meat, while in Germany, Australia, and Argentina, there is greater flexibility, provided that the labeling includes clear expressions such as “100% plant-based” or “made from plants.” Saudi Arabia and the European Union call for clarity and transparency, highlighting the importance of avoiding ambiguities regarding the plant-based origin of products [28,67].

Brazil and the European Union focus on clarity in the disclosure of information. Labels must be explicit and incorporate precise terms such as “plant-based” or “does not contain meat.” In this context, regulating the labeling and formulation of these foods is critical to guarantee consumer confidence. Meat replacements lack consistency and exhibit varying contents, complicating the comparison of their nutritional value to that of the animal-based products they aim to replace [27].

Brazil and South Korea are currently developing legislation for these products. In Brazil for example, Ordinance No. 1176/2024 from MAPA, published in September 2024 and submitted for public consultation, aims to define minimum requirements for identity, quality, labeling, and visual identity for meat analogs. The process aims to include organizations and civil society in the debate and provide information to support more conscious and healthy consumption choices [49].

**Figure 3:**
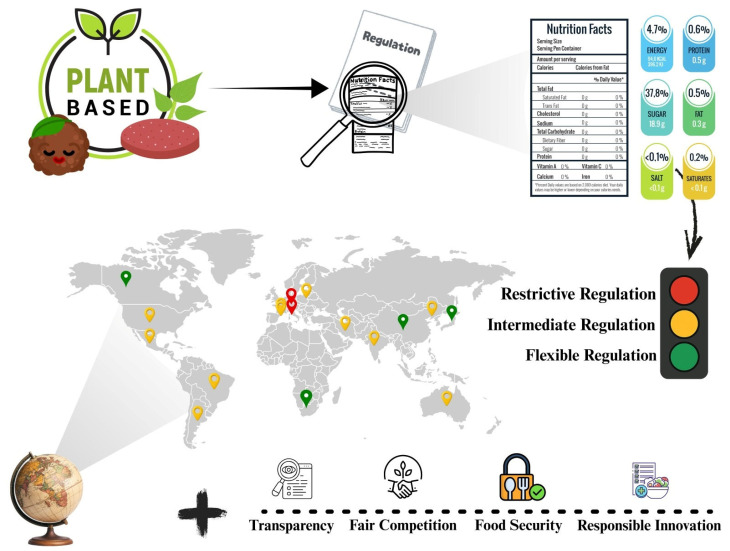
Global overview of regulatory approaches applied to meat analog products, highlighting countries with restrictive, intermediate and flexible regulations. **The color used above for each country, indicates the level of Regulation**. Source: Prepared by the author (2026).

Regulation of these items is necessary for a variety of reasons, as it ensures food and nutritional credibility and safety for consumers, mitigates the risk of errors, and enhances information transparency [26]. Musa-Veloso and Juana [27] state that governments act as an instrument of public policy that can align health, sustainability, and international commerce goals. For the industry, it promotes legal certainty and equitable competition, providing fair market conditions and encouraging responsible innovation. Figure 3 shows the present global framework of meat analog regulation, emphasizing nations with restrictive, intermediate, and flexible regulations, besides demonstrating the variety of regulatory strategies implemented globally.

According to Kumar et al. [68], the standardization of regulations at the global level could facilitate trade and scientific cooperation, as well as enable the standardization of technical and nutritional criteria. Standardization is particularly important considering the expansion of the alternative protein market and the sustainability demands identified by Laureati et al. [69].

Currently, countries with greater purchasing power have been investing in the development and regulation of meat analogs as alternatives to animal-derived proteins, with the stage of this evolution varying according to the context of each country [70]. However, government oversight and regulation of alternative proteins have not kept pace with business innovation [43].

Canada, China, Japan and South Africa are often mentioned as examples of success in the regulation of meat analogs. Canada and South Africa establish minimum nutritional parameters and require the use of the term “simulated” on packaging. China, in turn, promotes the reduction of sodium and fat, in addition to setting limits for proteins, vitamins, and minerals. Japan sets a minimum protein composition and requires clear labeling, using expressions like “soy food similar to meat” [26]. These practices represent well-balanced models, since they guarantee consumer reliability without limiting technological progress.

Advances have been observed in many countries, however political, economic, and cultural barriers continue to hinder the implementation of specific regulations. The traditional livestock and agribusiness sectors are showing resistance in various contexts, motivated by concerns about competition and the redefinition of the terms “meat” and “protein” [70]. The lack of international agreement on terminologies and nutritional parameters has prevented the development of standardized guidelines [71].

A considerable challenge is the disconnection between innovation and regulation. As companies invest in disruptive technologies, such as cultivated meat, legal frameworks remain outdated, resulting in legal insecurity and restrictions for international trade [43]. Successful experiences, such as those in Japan and Canada, could be a reference for the development of more inclusive and adaptable regulatory models [72].

The success of an evolving food product depends on the perception and acceptance of the public. In this sense, the presentation of these products must include communication and assertive messages so that the public can understand and accept these alternative proteins, in addition to the role of science in the creation of optimized products [73].

Considering this context, the international community has recognized the urgency of transformative changes to establish new paths for sustainable development and alternative protein sources [43].

Monaco [43] and Lähteenmäki-Uutela [42] mention that the Regulations applicable to Novel Foods are directly related to the protection of public health, consumer safety, and the proper functioning of the market. The main purpose was to guarantee that only foods that have been previously evaluated and considered safe are offered to consumers.

In this context, Monaco [43] also points out that, before a novel food or food ingredient can be marketed, the product must undergo an authorization process based on a scientific risk analysis. This analysis is conducted in a systematic and evidence-based manner, considering aspects such as safety, composition, nutritional value, allergenic potential, and conditions of use of the product.

On the other hand, Demartini et al. [41] emphasize that the absence of minimum global parameters regarding the identity, nutritional composition, and nomenclature of meat analogs can generate insecurity, hinder international trade, and negatively affect consumer confidence. Table 4 summarizes the main regulatory dimensions of meat analog products, with evidence of the challenges described in literature and their implications for consumption and regulatory frameworks.

As shown in Table 4, there are several challenges to be overcome with regard to the regulation of meat analogs. Among the challenges identified are the lack of standardized criteria for the nomenclature and definition of these products, the lack of clarity in labeling, the wide variability in their nutritional composition, and the mismatch between regulatory developments and technological innovations. These obstacles create uncertainty for consumers, jeopardize informed decisions, affect safety and confidence in the sector, and impose barriers to regulatory oversight, technological advancement, and international trade. This makes the need for greater regulatory alignment between countries even more necessary.

The regulatory aspects are necessary because many ongoing changes in eating habits have been observed, notably a decrease in meat consumption and a growing preference for diets predominantly based on plant-based foods.

## 5. Study Limitations and Future Perspectives

Several important limitations were identified in carrying out this study, which should be taken into account. First, the comparison between different levels of regulatory protection was based solely on information available in official documents, current legislation, public consultations, and scientific literature. Thus, the depth of the analysis depends directly on the degree of openness, up-to-date status, and details of each country’s regulations. In many cases, especially where there are no specific standards for products that mimic animal meat, the available information is fragmented, generic, or restricted to general food legislation, which substantially limits the scope and detail of the comparison between different international contexts.

Another relevant limitation is the absence of consolidated regulatory frameworks in most of the countries analyzed. This gap prevented the establishment of uniform criteria for comparing different national approaches, especially in relation to product identity, nutritional composition, nomenclature, and labeling requirements for meat analogs. The regulatory diversity observed makes it difficult to extrapolate the results, reinforcing the descriptive and exploratory nature of this research.

Additionally, it should be highlighted that the regulatory landscape for meat analogs is constantly evolving. The data presented reflects the current situation during the analysis period, which may undergo rapid changes, given that several countries are in the process of reviewing, conducting public consultations, or drafting new specific regulations for these products.

Based on this overview, as future perspectives for our research group, we are also working on plant-based beverages, with which the same concerns have been raised. Global recommendations and guidelines on plant-based milk are also being discussed and are increasingly necessary as consumption of these products and concern for sustainability grow. It is believed that recommendations for plant-based milk can vary according to each country and organization as well. The main focus of global health guidelines should be to guide consumers to understand that the chemical composition can vary considerably, considering the variety of plants (nuts, seeds, cereals, pseudo-cereals, legumes, vegetables, and fruits) of combinations that can be used in these products, their peculiarities in attempting to mimic the technological and sensory characteristics of animal milk, and how to use them in a healthy way in the diet.

## 6. Final Considerations

The analysis of the current landscape shows that meat analogs constitute one of the main innovations in response to the growing global concerns related to health, the environment, and animal welfare. These foods, primarily produced from plant-based proteins, emerge as relevant alternatives in response to the need to reduce the environmental impacts of livestock farming, which is responsible for significant greenhouse gas emissions, water consumption, and extensive land use.

The consolidation of these products in the market faces considerable challenges. Among them, the perception of unnaturalness, food neophobia, high prices, and the lack of nutritional and regulatory standardization stand out. At the same time, it is observed that consumers from different regions prioritize concerns such as flavor, sustainability, and economic accessibility, reinforcing the need for technological advancements and incentive policies to scale up production and reduce costs.

From a regulatory standpoint, the absence of universal standards reflects tensions between consumer protection, technological innovation, and commercial practices. While countries like France, Italy, and South Africa adopt restrictive stances regarding the use of terms traditionally associated with meat, other nations, such as China, Japan, and Germany, allow more flexible nomenclature, provided it is associated with technical and labeling requirements that ensure clarity and authenticity. In this context, international organizations such as ISO 23662:2021 and Codex Alimentarius [45] play an essential role, although they still have a lack of standardization in terms of nutritional equivalence and terminology.

The global trend of expanding the alternative protein market, driven by expressive investments and increased consumer acceptance, suggests that these products may soon achieve competitiveness in terms of commercial value. However, the success of this transition will depend on regulatory harmonization, the improvement of the nutritional profile, and overcoming the cultural and perceptual barriers that still limit its widespread adoption.

Thus, meat analogs represent not only a technological alternative but also a political, economic, and cultural challenge. The establishment of a clear regulatory environment, combined with investment in innovation and consumer knowledge, will be crucial for these foods to play a strategic role in achieving more sustainable, safe, and equitable food systems.

In this sense, it is advisable that public policies move towards the creation of regulatory frameworks capable of standardizing nutritional content, transparency of information, stimulating innovation, and accessibility to meat analogs. At the same time, policies that encourage research, technological development, and expansion of production scale are essential for advancing the consumption transition. For this to happen, public policies on food education and scientific communication are fundamental to reduce cultural and perceptual barriers to meat analogs.

## Figures and Tables

**Table 1:** Terms used for literature searching.

(“meat substitute*” OR “meat substitutes*” OR “meat analog*” OR “meat analogue*” OR “meat alternative*” OR “meat alternatives*” OR “meat-free product*” OR “meat free product*” OR “simulated meat*” OR “meat-like” OR “meat-like product*” OR “plant-based” OR “plant based” OR “plant-based meat*” OR “vegetable protein*” OR “non-animal protein*” OR “non animal protein*” OR “alternative protein*” OR “unconventional protein*” OR vegan*)
“Meat Substitutes” [Mesh] OR “meat analog*” OR “meat analogue*” OR “meat substitute*” OR “meat alternative*” OR “plant-based meat*”) AND (“Legislation, Food” [Mesh] OR “Food Standards” [Mesh] OR legislat* OR law* OR regulat* OR “food regulation*” OR regulament*)
(“meat analog*” OR “meat analogue*” OR “meat substitute*” OR “meat alternative*” OR “meat-free product*” OR “plant-based meat*” OR “simulated meat*” OR “meat-like product*”) AND (“food packaging” OR packaging OR label* OR labelling OR labeling OR “nutrition label*” OR “nutrition labelling” OR “nutritional inform*” OR “nutrition facts” OR “nutritional facts” OR “nutrition declaration” OR “front-of-pack*” OR “front of pack”)
(“meat analog*” OR “meat analogue*” OR “plant-based meat*” OR “meat substitute*”) AND (label* OR “nutrition label*” OR “nutritional inform*” OR “nutrition facts”)
(“Meat Substitutes” [Mesh] OR “Plant-Based Diet” [Mesh] OR “meat analog*” OR “meat substitute*” OR “plant-based meat*”) AND (“Food Consumption” [Mesh] OR “Consumer Behavior” [Mesh] OR “Feeding Behavior” [Mesh] OR “consumer trend*” OR “consumer perception*” OR “purchase intention*”)

Source: Study data.

**Table 2:** Consumption changes associated with the adoption of plant-based diets.

References/Year/Country	Studies	Consumption Indicator	Key Findings
Sullivan et al. [34] United States	Cross-sectional series (National Health and Nutrition Examination Survey 1999–2020; n = 51,698).	Time-based evolution of plant-based diets (≥50% plant protein) according to 24 h dietary recall.	The proportion of adults in the United States consuming plant-based diets increased from 14.4%. The quality of plant-based diets, as measured by Healthy Eating Index (HEI)-2020 scores, improved from 52.1% to 55.8%.
Strässner et al. [35] Germany	Validation/scale studies with consumers.	Behavioral changes to reduce meat consumption and adopt plant-based diets.	The study presents evidence on the effectiveness of a new Power Dialogue (PD) scale in reducing meat consumption, supported by psychometric validation. The research identified two superior factors and five inferior factors, suggesting that highlighting the benefits of a plant-based diet and minimizing perceived disadvantages may be effective strategies for motivating consumers to reconsider their eating habits in relation to meat. Among the barriers are health, legitimacy, and viability barriers of plant-based products.
Rizzo et al. [36] Italy	Consumer study (focus on habits)	Consumption of plant-based meat alternatives	The survey was conducted with people over the age of 18 and had 1142 participants. Among eligible participants (n = 874), 176 (20.14%) reported consuming these products at least two to three times a week. The survey emphasized the importance of public policies, marketing strategies, and labeling related to plant-based products. The results indicate that these dimensions play a key role in how consumers perceive and select plant-based products.
Jeżewska-Zychowicz et al. [37] Poland	Survey with adults (n = 1003)	Demonstration of interest in reducing meat consumption and increasing plant intake.	The regular frequency of plant-based food consumption, the negative feelings toward meat, and the environmental identity stimulated the consumer’s intention to consume more plant-based foods and less meat. On the other hand, the pleasure of eating animal meat was reduced.
Ford et al. [38] United Kingdom	Focus groups (young consumers)	Current habits: reduction in meat consumption and use of alternative proteins	Reducing and/or eliminating meat consumption was the most frequently mentioned change in eating habits, reported by 63% of participants. They increased their consumption of plant-based foods as meat substitutes. Some participants mentioned that nutritional values are considered a problem for full adherence to a plant-based diet, but it was mentioned that it would be easier to reduce meat consumption since there are some very satisfactory substitutes on the market.
Tan et al. [39] Switzerland	Argentina, Brazil, Canada, Chile, China, Colombia, France, Germany, India, Indonesia, Italy, Japan, Malaysia, Mexico, Netherlands, Poland, Russia, Singapore, South Korea, Spain, Thailand, United Kingdom, and United States (n = 20,966).	Relationship between meat consumption and familiarity/satisfaction with plant-based products	Moderate meat consumers have greater knowledge and satisfaction with plant-based products. Variations were observed between countries, with factors such as age and meat consumption having less influence on the acceptance of these foods in Asian countries, as plant-based products are part of local food traditions. These results are relevant to better understand the factors influencing the consumption of plant-based foods and identifying effective strategies to promote plant-based diets worldwide.

Source: Study data.

**Table 3:** Countries without specific regulations for meat analogs.

Country	Documents	Strategies and Action Plans	Authors/Year
**Argentina**	Joint Resolution of the Secretary of Health Quality and the Secretary of Food, Bioeconomy, and Regional Development No. 5/2022	Nomenclature: The use of official designations for animal-derived foods is prohibited, except to indicate aroma or flavor, and it is permitted to use terms that merely evoke them. Labeling: Products without animal-derived ingredients can display labels such as “100% plant-based,” provided that this indication appears prominently on the main face, alongside the product name.	Republic of Argentina [47]
**Australia**	Food Standards Australia New Zealand (ANZFS)—Information Requirements—Warning Statements, Advisory Statements, and Statements/2021	Nomenclature: It is mandatory to declare any other name by which this food is known. Labeling: Any food sold must include the phrase “based on a protein substitute” on the front of the packaging.	Australia [48]
**Brazil**	Final version of analogous products (in public consultation until 24 September 2024) SDA MAPA Ordinance No. 1176, 4 September 2024.	Nomenclature: The labeling must be clear, precise, and in Portuguese, in accordance with the legislation. Labeling: On the main panel, use “Vegetal analog of [name of animal product]”, accompanied by the phrase “this product does not replace the corresponding animal origin product”. It is prohibited to claim sustainability, health, GMO-free, natural, or organic, except as provided by law or proven.	Brazil [49]
**European Union**	Food Information for Consumers (European Union) No. 1169/2011	Labeling: The information on the packaging should not mislead consumers, and it should not be ambiguous or confusing.	European Union [50]
**France**	Decree nº 2022-144	Nomenclature: Prohibits the marketing of plant-based products labeled with terms related to meat and meat products, such as “sausage,” “steak,” and “bacon”; it also prohibits the use of terms like “ham,” “meat,” “fillet,” and “loin” to designate plant-based products.	France [51,52]
**Germany**	FIC (UE) n.º 1169/2011	Nomenclature: Terms such as “meatball” or “hamburger” can be used if there is a resemblance to the meat product. Labeling: “Vegetable meats” can only be included after a positive sensory test for appearance, taste, texture, and aroma.	Eurofins [53] Garcia et al. [28] Brazil Ikigai Law [54]
**India**	Food Safety and Standards Authority of India	Manufacturing: The production of vegan foods must occur in places free of non-vegan substances; when there is shared production line usage, complete cleaning is mandatory to avoid cross contamination. Labeling: It must comply with safety and food standards and display the approved vegan logo on the front of the packaging.	Food Safety and Standards Authority of India [55]
**Indonesia**	Regulation 25/2021 Regulation 31/2018	Labeling: Packaged products, including meat substitutes, must have clear labels with ingredients, nutritional information, and allergens. In meat substitutes, the absence of animal meat and the list of additives and preservatives must be indicated.	Indonesia [56,57]
**Italy**	Law 172/2023	Nomenclature: Prohibits the use of names, terms, or references related to meat, animal species, anatomy, butcher shops, delicatessens, fish markets, or animal-derived foods in non-meat products.	Italy [58]
**Mexico**	Official Mexican Standard 051-SCFI/SSA1-2010	Nomenclature: Allows the use of additional terms to avoid consumer deception. Imitation products must display “imitation” prominently in the upper left corner of the label. The term is not allowed on items with a protected designation of origin or a recognized geographical indication.	Mexico [59]
**Russia**	The country reported on regulations and standards for the import of food and agricultural products.	No regulations were found for meat analogs. The labeling rules are governed by general food legislation.	Ayala [60]
**Saudi Arabia**	Saudi Food and Drug Authority—FD/GSO 9: 2013-2013	Nomenclature: Products that imitate meat must contain clear information to avoid confusion with animal-origin products. Labeling: The information on the label must be presented in Arabic.	Saudi Arabia [61]
**South Korea**	Ministry of Food and Drug Safety	Updates on the establishment of legislation for alternative protein sources, including definition, specification, labeling, and safety assessment of technologies, including cultivated meat, should be implemented in 2024.	Neo [62]
**Turkey**	Food Code—2020	No specific regulation for meat analogs. The Codex restricts products that give the impression that cheese is produced with vegetable oil or other food ingredients.	Turkey [63]
**United Kingdom**	British Standard Institution—Public Specification Available 224/2020	Formulation: For those formulated with whole grains, nuts, seeds, and fruits, the use of any ingredient or product of animal origin is restricted.	British Standards Institution [64]
**USA**	Real Marketing Comestible Artificially Truthfully Act of 2019.	Nomenclature: meat imitation products can only be sold if the label prominently features the word “imitation” alongside the name and declares that they do not contain meat. Alternative foods are defined as “special dietary and nutritional additives,” including “plant-based edibles,” “soy-based,” or “plant protein products.”	United Nations. [65] United States Congress [66]

Source: Study data.

**Table 4:** Regulatory dimensions, main challenges, and implications for consumption and regulatory frameworks of meat analog products according to the literature.

Regulatory Dimension	Main Challenges Identified	Implications for Consumption Standards and Regulatory Frameworks	References/ Year/ Country
**Nomenclature and denomination of the product**	Lack of standardized criteria for the use of expressions referring to meat analogs products.	Consumer confusion, lack of safety, barriers to international trade, and declining market confidence.	Demartini et al. [41], Italy. Zhang et al. [26], China. Musa-Veloso & Juana [27], Canada. Seehafer & Bartels [60], Germany Caputo et al. [71], United States
**Labeling transparency**	General guidelines for food labeling often show inadequacies for new categories of meat analog products.	Misconceptions regarding the equivalence of meat analog products in relation to animal meat can compromise the intentional choice of consumers.	Lähteenmäki-Uutela et al. [42] Finland Pereira et al. [28] Brazil. Aschemann-Witzel et al. [8], Denmark. Laureati et al. [62], Italy.
**Product identity and definition**	Lack of defined criteria determining what characterizes meat analog products.	Difficulty in comparing products, regulatory oversight challenges, and limitations on innovation.	Zhang et al. [26], China. Monaco [43], Germany. Kumar et al. [68], India. Karmaus & Jones [73], United States.
**Nutritional composition and claims**	High variety in the ingredients of the formulations and lack of important nutritional parameters.	Risks associated with misleading health claims, nutritional imbalance and security concerns.	Lima et al. [40] Brazil. Shireen & Wright [24] Canada.
**The speed of Regulation in contrast to technological innovation**	Regulatory frameworks progress more gradually than technological innovation.	Insecurity and slowness in accessing the market, in addition to differences in regulatory protection among nations.	Monaco [43], Germany. Karmaus & Jones [73], United States.

Source: Study data.

## Data Availability

No new data were created or analyzed in this study.
